# S175. AMOTIVATION IS ASSOCIATED WITH SMALLER VENTRAL STRIATUM VOLUMES IN OLDER PATIENTS WITH SCHIZOPHRENIA

**DOI:** 10.1093/schbul/sby018.962

**Published:** 2018-04-01

**Authors:** Fernando Caravaggio, Gagan Fervaha, Yusuke Iwata, Eric Plitman, Jun Ku Chung, Shinichiro Nakajima, Wanna Mar, Philip Gerretsen, Julia Kim, Mallar Chakravarty, Benoit Mulsant, Bruce Pollock, David Mamo, Gary Remington, Ariel Graff-Guerrero

**Affiliations:** 1 Centre for Addiction and Mental Health, University of Toronto; 2 Queen’s University Faculty of Medicine; 3 Centre for Addiction and Mental Health; 4 McGill University

## Abstract

**Background:**

Motivational deficits are prevalent in patients with schizophrenia, persist despite antipsychotic treatment, and predict long‐term outcomes. Evidence suggests that patients with greater amotivation have smaller ventral striatum (VS) volumes. We wished to replicate this finding in a sample of older, chronically medicated patients with schizophrenia. Using structural imaging and positron emission tomography, we examined whether amotivation uniquely predicted VS volumes beyond the effects of striatal dopamine D2/3 receptor (D2/3R) blockade by antipsychotics.

**Methods:**

Data from 41 older schizophrenia patients (mean age: 60.2 ± 6.7; 11 female) were reanalysed from previously published imaging data. We constructed multivariate linear stepwise regression models with VS volumes as the dependent variable and various sociodemographic and clinical variables as the initial predictors: age, gender, total brain volume, and antipsychotic striatal D2/3R occupancy. Amotivation was included as a subsequent step to determine any unique relationships with VS volumes beyond the contribution of the covariates. In a reduced sample (n = 36), general cognition was also included as a covariate.

**Results:**

Amotivation uniquely explained 8% and 6% of the variance in right and left VS volumes, respectively (right: β = -.38, t = -2.48, P = .01; left: β = -.31, t = -2.17, P = .03). Considering cognition, amotivation levels uniquely explained 9% of the variance in right VS volumes (β = -.43, t = -0.26, P = .03).

**Discussion:**

We replicate and extend the finding of reduced VS volumes with greater amotivation. We demonstrate this relationship uniquely beyond the potential contributions of striatal D2/3R blockade by antipsychotics. Elucidating the structural correlates of amotivation in schizophrenia may help develop treatments for this presently irremediable deficit.

